# Role of the Growth Hormone Receptor (GHR) Gene in Skeletal Class II Malocclusion and Its Significant Influence on the Skeletal Facial Profile in Both the Sagittal and Vertical Dimensions: A Systematic Review

**DOI:** 10.7759/cureus.53596

**Published:** 2024-02-05

**Authors:** Ashwin Mathew George, A. Sumathi Felicita, Vijayashree J Priyadharsini, Anita P, Prasanna Aravind TR

**Affiliations:** 1 Orthodontics and Dentofacial Orthopaedics, Saveetha Dental College and Hospitals, Saveetha Institute of Medical and Technical Sciences, Saveetha University, Chennai, IND; 2 Clinical Genetics, Saveetha Dental College and Hospitals, Saveetha Institute of Medical and Technical Sciences, Saveetha University, Chennai, IND

**Keywords:** genetic resources, genetics, genotype, ramal height, mandibular body length, single nucleotide polymorphism, genetic polymorphism, ghr gene

## Abstract

This systematic review aims to determine the role of the growth hormone receptor (GHR) gene in skeletal malocclusion and its significant influence on the growth of the maxilla and the mandible in both sagittal and vertical dimensions. A search of the electronic databases of PubMed, Google Scholar, and Cochrane up to and including the year 2023 was made. In addition to this, a hand search of orthodontic and dentofacial orthopaedic journals was carried out. This search included randomized control trials. The Mesh terms used were “skeletal class II malocclusion”, “mandibular retrognathism”, “sagittal malocclusion”, “genetic expression”, “genetic factors”, "genetic study”, “genetic polymorphism”, and “single nucleotide polymorphism”. The inclusion criteria included studies such as clinical trials and orthopaedic appliances in the presurgical phase. The exclusion criteria for the study were studies not in the English language, case reports, case series, and studies with irrelevant data. It has been cited in various literature that polymorphic variations of the GHR gene could cause variations in mandibular morphogenesis affecting both the mandibular body length and ramal height. However, its effects are quite variable and are based on different population groups. Polymorphism of the GHR gene can be considered a reliable indicator predicting variations in affecting the growth of the mandible with greater significance in affecting the vertical ramal height compared to the body length of the mandible. Its effects on the maxillary skeletal base are rather limited comparatively.

## Introduction and background

Although there is a significant hereditary component to craniofacial shape, it is also altered by environmental variables, making research into it difficult. Many components of the head and neck region are involved in the normal development of the occlusion. Both the size of the maxilla and the mandible in the sagittal and vertical directions are the factors that determine the relationship between the two skeletal bases.

The linear and angular growth of craniofacial structures is significantly impacted by growth hormones (GH), which play a crucial part in the development of the craniofacial complex, resulting in malocclusion, which can be detrimental to the structural balance, aesthetics harmony, and stability of the dentition. This impact is both direct and indirect, making it essential to understand the role that GH plays in this process. Our understanding of the genetic process behind the development of maxillomandibular structures has significantly improved in recent years. As a result, we now have a better grasp of the potential morphological and functional abnormalities that may arise from these changes.

Single nucleotide polymorphisms (SNPs) and mutations are examples of genetic variations. Millions of SNPs can be possessed by an individual, and they are created by modifications in single nucleotides throughout the DNA sequence. These alterations are distributed for about every 100-300 bases. These genetic variants may increase or decrease the likelihood of developing a disease or result in a natural phenotype [1]. During early postnatal years, longitudinal growth and development are significantly influenced by GH. GH regulates cellular metabolism and differentiation and manages the metabolism of lipids, carbohydrates, and minerals. The growth hormone receptor (GHR) is the most significant binding protein that supports GH activity. GH is a peptide hormone that regulates craniofacial growth and development and is produced in the anterior pituitary gland.

The human GHR gene is located at 5p13.1-p12. The GHR gene, according to Godowski et al. [2], contains multiple extra exons in the 5-prime untranslated region in addition to the nine exons that encode the receptor. Chromosome 5's coding exons cover at least 87 kilobases. GHR gene expression is controlled by several variables, including development, diet, hormones, particularly GH, and tissue-specific factors. GHR gene polymorphisms and maxillomandibular characteristics have been demonstrated to significantly correlate in studies on a variety of regional groups, primarily Asians. Skeletal malocclusion could be caused by different factors such as mandibular retrognathism, maxillary prognathism, or a combination of both. In addition to the horizontal components of growth and development, vertical components such as variations in the ramal length and the inclinations of the maxillary and mandibular bases could affect the skeletal facial proportions. Treatment planning of skeletal malocclusion due to any of the above‑mentioned problems is age-dependent, considering the growth potential of the individual, before which the aetiology of malocclusion should be given top priority in planning the treatment and minimizing the relapse factor. Dentofacial orthopaedics done during the growth stage of an individual can successfully treat skeletal jaw discrepancies by modulating the growth of the skeletal bases of the jaws and face. However, when there is a genetic component involved in the aetiology of skeletal malocclusions, successful treatment with dentofacial orthopaedics becomes questionable. It is therefore important to analyze the genetic component contributing to skeletal malocclusion so that appropriate steps can be taken to decide on the optimum treatment plan.

An existing systematic review [3] has already evaluated the GHR gene polymorphism in different populations associated with Angle’s class III and mandibular protrusion relating to the mandibular body length. Apart from its effect on the mandibular body length, the GH plays a role in controlling the ramal as evaluated by Yamaguchi et al. in the Japanese populations [4]. Another factor of importance is the role of the GH and its effects on different ethnic populations. Therefore, this systematic review aimed to evaluate the role of the GHR gene polymorphism in skeletal malocclusion and its significant influence on affecting the growth of the maxillary and mandibular jaw in both the sagittal and vertical dimensions.

## Review

Materials and methods

**Figure 1:**
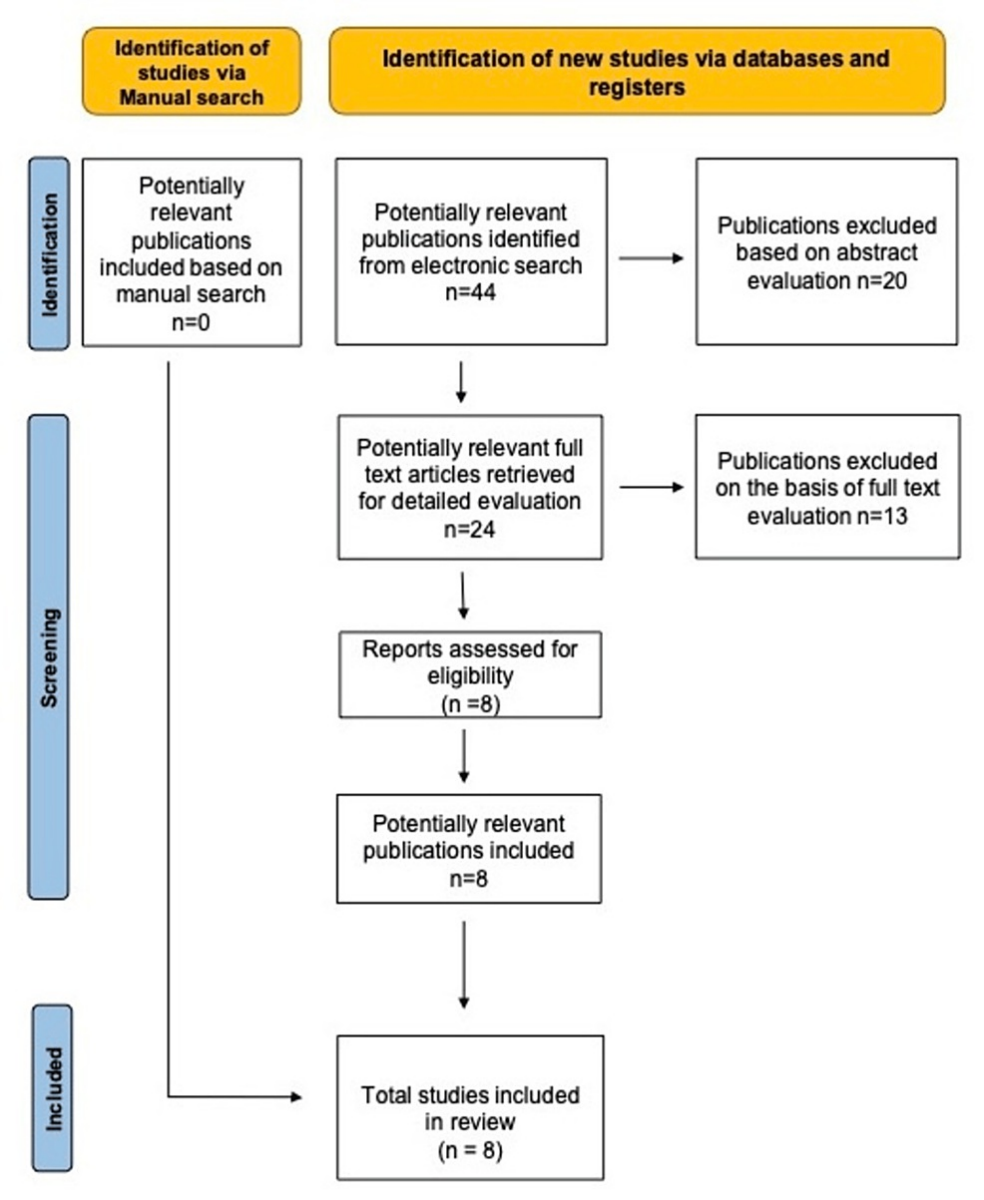
PRISMA flow chart of the articles evaluated for this study PRISMA - Preferred Reporting Items for Systematic Reviews and Meta-Analyses

An electronic database of PubMed, Google Scholar, and Cochrane up to and including the year 2023 was made. In addition to this, a hand search of orthodontic and dentofacial orthopaedic journals was carried out. Clinical trials and randomized control trials were included and studied. The Mesh terms used were “skeletal class II malocclusion”, “mandibular retrognathism”, “sagittal malocclusion”, “genetic expression”, “genetic factors”, "genetic study”, “genetic polymorphism”, and “single nucleotide polymorphism”. The inclusion criteria were studies including orthopaedic appliances in the presurgical phase and clinical trials. The exclusion criteria for the study were studies not in the English language and studies with irrelevant data. The PRISMA analysis (Figure 1) provides a full explanation of the search technique.

Eligibility criteria

The study was conducted to comply with the PRISMA guidelines. The study included original, cross-sectional, and case-control studies that evaluated the GHR gene's role in skeletal malocclusion and its impact on maxillary and mandibular growth in both sagittal and vertical dimensions. Manuscripts that were not published, theses, book chapters, case reports, and dissertations were removed.

Study selection

Two reviewers independently examined all the paper titles and abstracts. If one reviewer determined that the publication met the inclusion criteria, the complete text was obtained. The full-text analysis included abstracts that were potentially eligible, as well as those that did not provide enough information, were analyzed. Case-control studies did not meet the final selection criteria after a full-text evaluation because of a lack of homogeneity in the methodology. Hence, they were not involved in this systematic review. Any eligibility conflicts were addressed through consensus after analyzing the complete text, and if conflicts remained, a third reviewer was invited to make the final judgment. Additionally, the following details were collected: author/year, ethnicity/country, age range, sample size, case description, and authors' findings.

Qualitative assessment

The risk of bias for the included studies was performed using the Joanna Briggs tool for cross-sectional studies [5].

Result

Extracted data in this review are listed in Table 1. There were 44 potentially relevant publications that were identified from the electronic search, and 20 publications were excluded based on abstract evaluation. Of these 24, potentially relevant full-text articles were retrieved. After a precise evaluation, 16 publications were excluded, and eight publications were selected for final review.

**Table 1:** Articles meeting the inclusion criteria that were included in this study Sources: [4,6-12] SNP - single nucleotide polymorphism GHR gene - growth hormone receptor gene

Author	Objective	Research methodology	Population	Sample	Specimen	Outcome
Yamaguchi et al., 2001 [4]	This study was aimed at quantitatively evaluating the relationship between craniofacial morphology and the P56IT variant in the GHR gene.	Cross-sectional	Japanese	50 Japanese men (20-49 years) and 50 Japanese women (18-46 years).	Blood	The normal Japanese population without P56IT had a significantly greater mandibular ramus length (condyliongonion) than did those with P56IT. This suggests that the GHR gene P56IT variant may be associated with mandibular height growth and can be a genetic marker for it.
Zhou et al., 2005 [6]		Cross-sectional	Chinese	145 Han Chinese	Blood	Significant correlation was found between I526L and mandibular ramus height. Individuals with genotype CC had increased ramus lengths compared to those with genotype AC or AA, in the first population.
Sasaki et al., 2009 [7]	To determine whether the P561T heterozygous missense mutation affects mandibular growth during the early stages of growth and development in humans.	Cross-sectional	Japanese	33-mandibular protrusion.27 -normal children with a Class I occlusion	Briefly, buccal epithelia cells were collected by twirling a sterile cytology brush on the inner cheek for 30 seconds. DNA was extracted using a BuccalAmp™ DNA extraction kit (Epicentre, Madison, Wisconsin, USA) according to the manufacturer’s protocol	P561T heterozygous mutation did not account for the difference between mandibular protrusion and normal occlusion, but may have an effect on mandibular growth itself during early childhood, indicating that this mutation in the GHR gene functions as an inhibitory factor in the process of mandibular growth.
Tomoyasu et al., 2009 [8]	To find the association between mandibular height and the growth hormone receptor gene	Cross-sectional	Japanese population	167	Blood	Results indicate that the GHR polymorphisms P561T and C422F are associated with mandibular ramus height in Japanese population and suggest that the SNPs of the GHR associated with the Japanese are likely to be different in other ethnic group.
Kang et al., 2009 [9]	To characterize further the roles of the d3/fl-GHR SNP of GHR and five SNPs in exon 10 of GHR in 159 Korean subjects with regard to craniofacial morphology, and to define the allelic frequencies of d3/fl-GHR in a multiethnic population, which consisted of Han Chinese, African Americans, European Americans, and Hispanics.		Korean subjects Han Chinese, African Americans, European-Americans, and 24 Hispanics	159 Korean subjects 24 Han Chinese, 24 African Americans, 24 European Americans, and 24 Hispanics		Found a significant association between the P561T and C422F polymorphisms of GHR and mandibular ramus height in a Korean population.
Tobón-Arroyave et al., 2017 [10]	Examine the association between the rs6184 and rs6180 polymorphic variants of the growth hormone receptor (GHR) gene and skeletal-facial profile in a Colombian population.	Cross-sectional	Colombian population	306	Saliva	Allele A of rs6184 SNP alone or in combination with other SNPs in the GHR gene may account for significant horizontal and longitudinal variations of the mandibular morphology and might be a strong/independent prognostic indicator for Class III skeletal-facial profile in the population.
Nakawaki et al., 2017 [11]	Examine the relationship between three-dimensional mandibular morphology and growth hormone receptor (GHR) gene variants in a healthy Japanese population.	Cross-sectional	Japanese	64 men and 114 women	Saliva	Revealed that the rs6180 variant of the GHR gene is correlated with the distance between the left and right coronoid processes in the population studied.
Sameemullah et al., 2023 [12]	The aim of this study was to determine the association of single nucleotide polymorphisms (SNP) of GHR gene rs6180, rs6182, and rs6184 with maxillomandibular parameters in the Indian population.	Cross-sectional	Indian	174 male and female Indian subjects in the age range of 20-32 years	Blood	SNP rs6180 and rs6182 were associated with sagittal mandibular position and length, ramal length, anterior facial height, and growth pattern.

Polymorphisms in the GHR gene did result in an inhibitory effect on mandibular morphogenesis but showed more significance in affecting the mandibular height (ramal height) compared to the mandibular body length (corpus length). Among the various polymorphisms studied, it was P56IT of the GH gene that showed the greatest influence, especially in determining the mandibular body length and mandibular ramal height. However, when evaluating critically, it was noticed that the effects of the GH in determining craniofacial morphology vary vastly when comparing different ethnic populations. There was also a notable difference in the allele frequency with the GHR 1526L polymorphism in the Chinese population affecting the mandibular height with a variation observed in the genotype showing the CC genotype correlating with a longer ramus than those with the genotype AC or AA. Studies done in the Colombian population showed that the presence of allele A of rs6184 SNP, either alone or in conjunction with other SNPs in the GHR gene, could be an independent prognostic indicator primarily for Class III skeletal profiles. On the other hand, rs6180 did not show any significant alterations in mandibular morphology.

In the Indian population, it was identified that rs6180 showed a greater influence compared to rs6184 in affecting mandibular morphogenesis; however, these results were not statistically significant. Concerning the maxilla, SNP rs6180 is linked to the sagittal position of the maxilla, while SNP rs6182 is associated with its length. However, the genotypes of SNP rs6184 did not show any significant association.

Qualitative assessment

**Figure 2:**
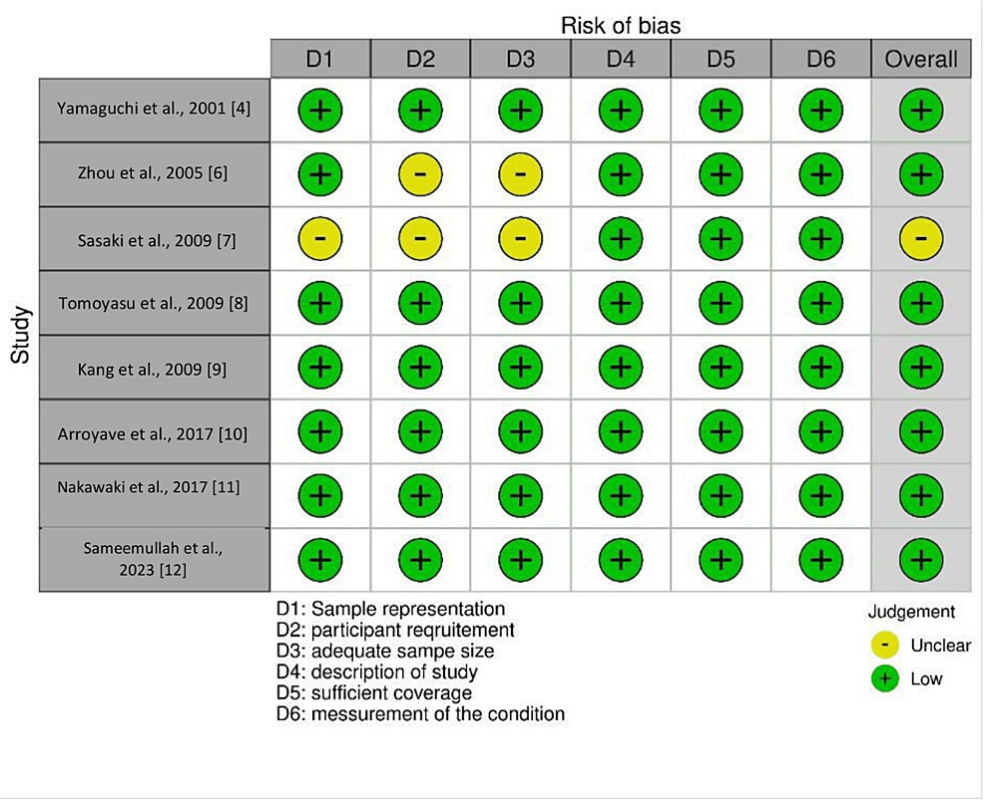
Risk of bias of the included studies Sources: [4,6-12]

Out of the eight included studies, all studies had a low risk of bias, except for the study by Sasaki et al. [7], which has some concerns with respect to sample representation, participant recruitment, and adequate sample size (Figure 2).

Discussion

Craniofacial growth is influenced by a variety of genetic, hormonal, and environmental factors. The GH contributes to bone homeostasis, with hormones, cytokines, and other local growth factors playing a major role in the same [13]. The anterior pituitary secretes the GH, which is a peptide, and they are regulated by the brain and can also be affected by different hormones such as glucocorticoids, sex steroids, and thyroid hormones [14]. Osteoblasts are stimulated by these hormones, and the pressure from muscles on bones leads to the growth of bone tissue, mainly affecting endochondral ossification in the condyle of the mandible and the cranial base. GH deficiency individuals have smaller craniofacial dimensions, including a reduced facial height and width, a head circumference being smaller, a protruding frontal bone, a saddle nose, and a convex profile, with a relatively reduced mandibular and maxillary length and manifesting a smaller cranial base length. Gene expressions are manifested and carried through the functions of the GH [15]. The gene encoding the GHR receptor, responsible for activating GH function, is located on chromosome 5's proximal short arm. It is essential to note that the GHR gene is present in almost every cell of the human body. Any modifications made to the receptor can result in severe repercussions, such as stunted growth, weakened muscle strength, decreased bone mineral density, thinning hair and skin, delayed onset of puberty, and an increased risk of obesity. Additionally, mutations in the GHR gene's extracellular domain may result in GH insensitivity caused due to loss of function. In a review by Frazer et al., he reported that individuals with GHR gene variants respond differently to GH [16]. Many studies have been carried out to evaluate the impact of GHR polymorphisms on craniofacial morphology in different populations [4-12] with many corroboratory and few contradictory results. Therefore, this systematic review was performed to understand the impact of GHR polymorphism causing variations in the normal skeletal development of the maxilla and mandible to derive some sort of conclusive evidence. Yamaguchi et al. [4] studied the relationships between the Pro561T variant of the GHR and craniofacial morphology in a sample Japanese population and discovered that the normal Japanese population without P56IT had a significantly longer mandibular ramus (condylion to gonion) than did the P56IT group, suggesting that the GHR gene P56IT variant may be linked to mandibular height growth and can be a genetic marker for it.

A study by Tomoyasu et al. [8] examined the possible association of five SNP variants, namely, S473S (rs6176), P561T (rs6184), P477T (rs6183), I526L (rs6180), and C422F (rs6182), and of the GHR gene at exon 10 with craniofacial morphology. The study was conducted among 167 subjects of the Japanese population. The major allele frequencies for four SNPs were found to be above 90% in the population studied. The distribution of the A and C alleles of the I526L polymorphism was almost equal. A multi-ethnic comparison was also performed in Han Chinese, European American, Hispanic, and African American populations. Interestingly, the minor allele frequency was almost nil for the Hispanic and African American populations for all SNPs, except I526L. The European American population analysis returned three SNPs with nil minor allele frequency. The variations in the allele frequencies among different populations and different ethnic groups substantiate the need to investigate further the genotype and allele frequencies in individual populations and to derive an association with unique phenotypes. Haplotype analysis confirmed that individuals with the combination of CC + GG genotypes had markedly greater mandibular ramus height when compared to individuals with the CA and GT genotypes. In addition, four of the five variants analyzed showed genotype frequencies exclusive to the Asian population.

To determine if there is an association between variations in GHR and craniofacial morphology among the Korean population, Kang et al. [9] showed that there was an association between P561T and C422F SNPs of the GHR locus in the Korean population and found associations between these SNPs and mandibular ramal height, similar to the sample Japanese population. However, in contrast to Zhou et al.'s [6] findings in Chinese populations, Kang et al. discovered no relationship between the I526L polymorphism and the mandibular ramal height.

Zhou et al. [6] investigated the relationship between mandibular height and GHR variations and discovered no association between C422F, G168G, or P561T with any of the measurement parameters contrary to Yamaguchi et al.'s findings. However, they found an association with the genotype AC or AA of the GHR 1526L variant, showing a shorter ramal length compared with the CC genotype.

A study was designed by Sasaki et al. [7] to assess the influence of a missense mutation of the GHR gene, P561T on mandibular growth in children. The subjects were recruited in the case or control category based on the cephalometric measurements. The case group included 33 children having a mandibular protrusion. The control group had 27 normal children. Genomic DNA was isolated from the buccal epithelial cells and subjected to genotype analysis based on the polymerase chain reaction (PCR) and restriction fragment length polymorphism (RFLP) protocol. Analyzing the study participants, five children with mandibular protrusion and two normal subjects were found to possess a heterozygous genotype. Although a statistically insignificant presentation, the multi-model analysis revealed that the mutation decreased the linear mandibular measurements, thus providing evidence of the possible consequence of the mutation on mandibular growth.

Tobón-Arroyave [10] investigated the correlation between the GHR gene's polymorphic alleles rs6184 and rs6180 with the skeletal profile of the face in the Colombian population and concluded that allele A of rs6184 SNP, alone or associated with others in the GHR gene SNPs, showed the possibility of significant variations in the horizontal and longitudinal dimensions of the mandibular morphology. The analysis was performed among 306 individuals who were categorized based on the skeletal facial profile into three groups, namely, Class I, Class II, or Class III. A PCR-RFLP-based approach was employed to identify the genotypes of individuals, followed by multivariate analysis. The study concluded that the allele A of rs6184 polymorphism could act as an independent measure for Class III malocclusion in the population studied.

Nakawaki et al. [11] have unequivocally established the correlation between GHR gene variants and mandibular morphology in a healthy Japanese population. The undisputed evidence demonstrates that the rs6180 GHR variant is incontrovertibly linked to variations in the distance between the left and right coronoid processes in this specific population.

The study conducted by Sameemullah et al. [12] in the Indian population revealed a clear association between the SNP of the GHR gene (rs6180, rs6182, and rs6184) and the skeletal maxillary and mandibular bases. Their findings demonstrate that SNP rs6180 and rs6182 play a crucial role in determining mandibular position in the sagittal plane, ramal length in the vertical plane, and the lower anterior facial height. This study shows that GHR polymorphisms influence mandibular and other cranial structural development. Numerous environmental factors can affect mandibular development, but genetic factors are also quite significant.

The studies listed above showed an association between polymorphisms in P561T and C422F of the GHR gene and mandibular vertical ramal height (condylion-gonion) in Korean and Japanese populations [4,9]. A significant correlation between the GHR mutation and mandibular ramal height was discovered in a study conducted on Chinese adults. However, it is important to note that the results varied across different populations, including Chinese, Japanese, and Korean. There was a connection between the polymorphic variations, rs6182, rs6184, and rs6180 in GHR and condylion-gonion measurements among the Asian population. However, they found little support for the above association between Colombian [10] and Egyptian populations. An understanding of the molecular mechanisms behind the growth of the mandible could be useful in both diagnosis and treatment planning for patients who would require growth modification procedures during orthodontic treatment.

A study conducted by He et al. [17], to determine the influence of gene polymorphisms on facial morphology, returned interesting results relating to the involvement of the GHR gene. The study was conducted in a large cohort of the Uygur population in China. About 578 volunteers were recruited in the study, and the saliva samples were collected. Ten different SNPs of four genes were investigated for possible association with facial morphology. Each of the GHR gene polymorphisms rs6180 and rs6184 was found to be associated with the soft tissue lip linear length and the lip curve, respectively. These studies provide ample evidence about the role of genetic variants in establishing a phenotypic trait. More insight into GHR gene polymorphisms in different ethnic populations could provide valuable clues about the influence of this gene in craniofacial development and other disorders associated with it.

It should be considered that genetic associations may be influenced by racial and ethnic differences. Research findings suggest that there is indeed more genetic diversity within continents than between them. To be more specific, the level of genetic variation within a continent can range from 85% to 90%. Meanwhile, the genetic variation between continents is only between 10% and 15%. This emphasizes the importance of understanding and studying genetic diversity within populations on a regional level [18]. With the rapid development of next-generation DNA sequencing technologies, it is expected that hundreds of thousands of novel human SNPs will be discovered in the coming years. Genome-wide association Study (GWAS) has also been utilized to identify disease-related SNPs that play a crucial role in comprehending the molecular mechanisms of evolution.

During childhood and adolescence, the growth hormone receptor (GHR) is absolutely crucial in regulating growth and metabolism by binding to specific receptors on cell surfaces [19]. The functional matrix theory was given by Melwin Moss who proposed that the stretch of the soft tissues results in modulating bony growth [20]. Therefore, the detection of any genetic disorders early in childhood affecting the growth of the facial skeleton could give the clinician an insight to take advantage of the growth potential. Measures to modify growth by activating the muscle of the facial skeleton using functional appliances could prove successful.

The influence of GHR polymorphisms was investigated in several other phenotypes relating to mandibular morphology. One such study conducted by Park et al. [21] and the team demonstrated the association of three polymorphisms, viz., rs6180, rs6182, and rs6184, with mandibular prognathism. Although the association was found to be insignificant, the rs6180 polymorphism was found to exhibit a marked association with that of SNMP, ANB, and SNB and effective mandibular length in females. Interestingly, rs6182 and rs6184 were found to be associated with ramal height in males. Thus, this study deciphers the uniqueness of GHR SNPs investigated with each phenotypic trait that influences the skeletal facial profile.

The greater the genetic contribution to malocclusion, the lesser the success rate of orthodontic and orthopaedic treatment. The success of combined orthodontic and orthopaedic treatment for growth modulation of the maxillary and mandibular bases largely depends on the patient's genetic potential. Any SNP or mutations found in the genes responsible for the growth of the maxilla and mandible could be detrimental to the overall facial appearance. In this regard, genotype studies could aid in the diagnosis and treatment planning by gauging the craniofacial growth potential. Identifying the major genes and their impact on skeletal discrepancies is crucial for making progress in this area. To achieve this, genetic studies are necessary to pinpoint the specific genetic markers linked to certain dental or skeletal malocclusions. Although the most definitive correct treatment modality should be the modification of the gene responsible for maxillary prognathism or mandibular retrognathism whichever is the cause, it is currently just a hypothetical proposal.

## Conclusions

The polymorphism in the GHR gene can be used as a reliable indicator in predicting the variations in the growth of the maxilla, mandible, and other craniofacial structures. However, conclusive evidence cannot be ascertained as the results of this systematic review showed that different populations showed variations in the genotype and allele frequency that resulted in the varied expression of the GHR gene. Because of the lack of generalizability of the results, population-specific analysis of SNPs of the GHR gene needs to be further evaluated, which would add to the validity of existing studies.
